# Association Between Gut Microbiota and Chronic Kidney Disease: A Two-Sample Mendelian Randomization Study in a Chinese Population

**DOI:** 10.3390/biomedicines13061397

**Published:** 2025-06-06

**Authors:** Wenjian Lin, Zixin Liang, Junxuan Fang, Yu Liu, Lei Lei, Jiawen Lin, Bin Xia, Zhihua Zheng, Jingqiu Yuan, Chun Tang

**Affiliations:** 1 Department of Nephrology, Center of Kidney and Urology, The Seventh Affiliated Hospital, Sun Yat-Sen University, Shenzhen 518107, China; linwj25@mail2.sysu.edu.cn (W.L.); fangjx8@mail2.sysu.edu.cn (J.F.); liuy965@mail2.sysu.edu.cn (Y.L.); leilei35@mail.sysu.edu.cn (L.L.); linjiaw3@mail2.sysu.edu.cn (J.L.); zhzhihua@mail.sysu.edu.cn (Z.Z.); 2 Department of Epidemiology and Biostatistics, Clinical Big Data Research Center, The Seventh Affiliated Hospital, Sun Yat-Sen University, Shenzhen 518107, China; liangzx9@mail2.sysu.edu.cn (Z.L.); xiab7@mail.sysu.edu.cn (B.X.); 3 Chinese Health Risk Management Collaboration (CHRIMAC), Shenzhen 518107, China

**Keywords:** chronic kidney disease, gut microbiota, Mendelian randomization analysis, mediation analysis, serum proteins

## Abstract

**Background:** Population differences in gut microbiota composition and related metabolites may influence their potential causal relationship with chronic kidney disease (CKD); however, this relationship remains poorly understood in the Chinese population. **Materials and Methods:** We conducted a two-sample Mendelian randomization (MR) study using summary statistics of 500 gut microbial features (9 phyla, 3 classes, 14 orders, 32 families, 95 genera, 248 species, and 99 gut metabolic modules (GMMs)) from the 4D-SZ (from Shenzhen, China) discovery cohort (n = 1539). CKD summary statistics were obtained from the China Kadoorie Biobank (CKB) (489 cases and 75,531 controls). Associations between gut microbiota and CKD were evaluated via inverse variance weighted, MR-Egger, weighted median, and MR-PRESSO. To validate our findings, we replicated the analyses in two independent East Asian CKD GWAS datasets: the Biobank of Japan (BBJ) dataset (2117 cases and 174,345 controls) and the J-Kidney-Biobank (JKB) dataset (382 cases and 3471 controls). We further validated the results via a meta-GWAS of BUN and eGFR in Biobank Japan (BBJ) and the Taiwan Biobank (TWB). Additionally, we analyzed 304 serum proteins from the Guangzhou Nutrition and Health Study (GNHS) and conducted mediation MR analyses to explore potential mediators. **Result:** At the locus-wide significance threshold, we identified 18 gut microbiome features associated with CKD onset in the China Kadoorie Biobank (CKB). Genus *Alistipes* (OR 1.02, 95% CI 1.00–1.03, *p* = 0.03) was associated with incident CKD risk in the JKB cohort. Species *Bifidobacterium catenulatum*–*Bifidobacterium pseudocatenulatum* complex (OR 1.0074, 95% CI 1.0070–1.0142, *p* = 0.01) was associated with incident CKD risk in a meta-GWAS of BUN. Sensitivity analyses, including Cochran’s Q test, MR-Egger intercept analysis, leave-one-out analysis, and funnel plots, yielded consistent results. Mediation analysis revealed that 26.7% (95% CI: 0.006–0.6700, *p* = 0.04) of the effect of *Alistipes* on CKD risk was mediated through the serum protein FBLN1. **Conclusions:** Our study provides Mendelian randomization-based evidence supporting a potential causal relationship between gut microbiota and CKD, highlighting the potential mediating role of FBLN1 in the association between genus *Alistipes* and CKD. Further studies are needed to explore whether and how genus *Alistipes* and FBLN1 contribute to CKD development.

## 1. Introduction

Chronic kidney disease (CKD), also known as chronic kidney failure (CRF), is one of the leading causes of death and morbidity in the 21st century, affecting more than 10% of the global population, more than 800 million individuals worldwide [1]. Although the mortality rate among patients with end-stage kidney disease (ESKD) has declined [2], studies from the Global Burden of Disease (GBD) have identified CKD as a significant contributor to global mortality [3,4]. Patients with CKD face elevated risks of all-cause mortality, cardiovascular-related death, and kidney failure requiring replacement therapy (chronic dialysis or kidney transplantation). They also experience higher rates of hospitalization due to complications such as stroke (both ischemic and hemorrhagic), myocardial infarction, heart failure (any hospitalization or death related to heart failure), acute kidney injury, atrial fibrillation, and peripheral artery disease [5]. This situation poses a substantial burden on patients, healthcare systems, and society. Numerous well-established risk factors for CKD—such as age [5], hypertension [6], obesity [7,8], diabetes [9], cardiovascular disease (CVD) [10], and hyperlipidemia [11]—are associated with significant alterations in the composition and function of the gut microbiome. However, the causal relationship between the gut microbiota and CKD remains poorly understood. Investigating the potential causal effects of the gut microbiota and identifying potential mediators could provide critical insights for mechanistic studies and inform clinical interventions for CKD management.

Randomized, double-blind, placebo-controlled trials have demonstrated that probiotic use is associated with reductions in serum blood urea nitrogen (BUN, a measure of kidney function) and uric acid levels, as well as improvements in quality of life [12,13,14,15,16]. However, the small sample sizes of these trials limit the applicability and generalizability of their findings. Observational cohort studies have suggested that gut microbiota dysbiosis may play a role in the development and progression of CKD, with differences observed between CKD patients and healthy controls, as well as across different stages of CKD [17,18,19,20,21]. Serum and fecal metabolomic analyses further support the functional role of the gut microbiota in CKD pathophysiology, linking it to both the onset and progression of CKD [17,18,19,20,21]. Nevertheless, these studies have reported inconsistent results regarding specific alterations in the gut microbiota composition in CKD patients compared with non-CKD individuals [17,18,19,20,21]. Experimental evidence indicates that certain gut microbiota components, such as *Bacteroides fragilis*, can alleviate renal fibrosis [22] and that short-chain fatty acids (SCFAs) exhibit protective effects against diabetic nephropathy [23]. Dysbiosis of the gut microbiome has also been associated with risk factors for CKD, including type 2 diabetes, obesity, hypertension, and heart failure [6,10,24,25].

Clinical research suggests that serum protein levels can predict the incidence of CKD [26,27], and the gut microbiome can directly or indirectly influence serum protein levels, thereby affecting disease onset. Emerging evidence highlights the complex interplay between genetics and the gut microbiome in regulating circulating proteins that modulate various biological processes, with effects observed across multiple organs and tissues [28]. Additionally, recent studies have identified a weak yet significant association between the gut microbiota and the total plasma N-glycome—the complete set of N-linked glycans attached to proteins circulating in human blood plasma, which reflect both genetic and environmental influences on glycosylation [29]. Moreover, serum proteins and circulating metabolites have been shown to mediate the potential causal effects of gut bacteria on osteoporosis development, further underscoring their role in disease mechanisms [30]. Despite these findings, observational studies are limited by confounding factors and reverse causality, making establishing causal relationships challenging. As a result, whether the gut microbiota exerts a potential causal effect on CKD and whether serum proteins or other risk factors mediate this effect remain largely unexplored.

Mendelian randomization (MR) is a methodological approach that uses genetic variants associated with a specific risk factor as instrumental variables to assess the potential causal effect of that exposure on a particular outcome. This method emulates the randomization process of randomized controlled trials. Large-scale genome-wide association studies (GWASs) have identified numerous single nucleotide polymorphisms (SNPs) linked to CKD, the gut microbiota, and serum proteins, offering a valuable opportunity to explore potential causal relationships between them through MR analysis. Recent studies have shown that the heritability of the gut microbiome is high, with microbial taxa exhibiting significant heritability in the TwinsUK cohort [31,32]. In fact, the composition of the gut microbiota varies significantly between different populations [33,34,35,36]. Although there are many Mendelian randomization studies [37,38,39] on gut microbiota and CKD, there is a lack of evidence regarding the relationship between gut microbiota and CKD in the Chinese population because these previous studies are based on European populations [37,39,40,41]. Furthermore, Mendelian randomization has been widely utilized to conduct mediation analyses [42,43,44], providing insights into the pathways through which an exposure affects an outcome. However, whether serum proteins mediate the causal relationship between gut microbiota and CKD remains unclear.

Fibulin-1 (FBLN1) is an extracellular matrix glycoprotein involved in cell adhesion and tissue remodeling. It has been implicated in various physiological and pathological processes, including myelofibrosis [45], cardiovascular risk development [46], and kidney function [47]. Recent studies suggest that FBLN1 levels are associated with renal fibrosis and impaired kidney function [46,47], yet its potential causal role in CKD has not been systematically explored. Moreover, it remains unclear whether FBLN1 serves as a mediator linking gut microbiota to CKD progression, representing a critical knowledge gap. Addressing this gap may uncover novel mechanisms and potential therapeutic targets for CKD. In this study, we investigated the causal relationship between the gut microbiota and CKD using a two-sample Mendelian randomization (MR) design within a Chinese population cohort. Our findings were replicated and validated in two independent datasets as well as in a meta-GWAS (a statistical approach that combines summary results from multiple genome-wide association studies to increase power and identify consistent genetic variants associated with a trait) of BUN and eGFR (a clinical measure of kidney function). Furthermore, we performed MR analyses to explore the associations among microbiome features, serum proteins, and CKD. Mediation MR analysis was subsequently conducted to evaluate the mediating role of CKD-related serum proteins in the relationship between gut microbiota and CKD. Our results indicate a potential causal link between specific gut microbiota and CKD, with several CKD risk factors serving as mediators in these associations.

## 2. Method

### 2.1. Study Design

**Figure 1:**
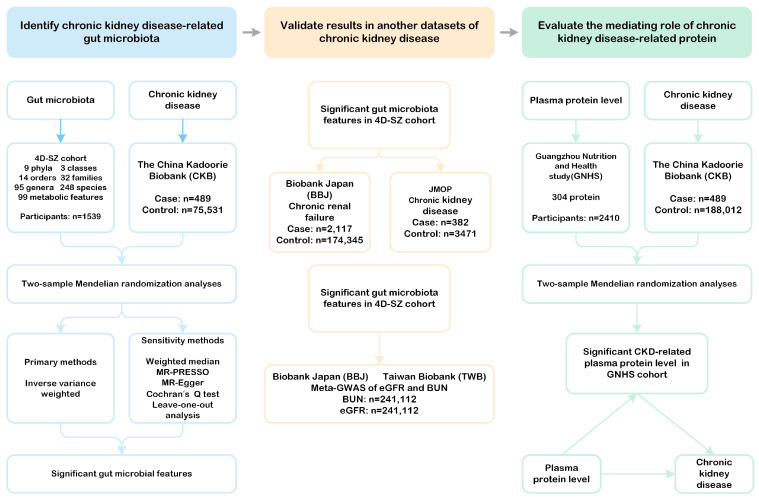
Study design. This flowchart illustrates the framework of our investigation, which is structured into three main phases. Phase 1 involved conducting a two-sample Mendelian randomization (MR) analysis utilizing the inverse variance weighted method along with multiple sensitivity analyses to identify potential causal gut microbial taxa linked to chronic kidney disease in the Chinese population. Phase 2 focused on validating the significant gut microbial taxa identified in the first phase by using two summary-level datasets related to chronic kidney disease and chronic renal failure, as well as meta-GWAS data for BUN and eGFR sourced from BBJ and TWB. Phase 3 comprised a mediation MR analysis, where we assessed the causal associations between gut microbial taxa and various serum proteins. Additionally, we determined the extent to which these microbial taxa influence atrial fibrillation through mediation by these risk factors. **MR-PRESSO:** MR Pleiotropy RESidual Sum and Outlier. **MR-Egger:** Mendelian randomization Egger regression. **BBJ:** BioBank Japan. **JMOP:** Japanese Multi-Omics Reference Panel. **eGFR:** estimated glomerular filtration rate. **BUN:** blood urea nitrogen. **GNHS:** Guangzhou Nutrition and Health Study.

The study design is illustrated in Figure 1. First, we conducted two-sample MR analyses to identify gut microbial taxa with potential causal effects on CKD in a Chinese population. Next, we validated these findings using summary-level data via two independent cohorts: the Biobank of Japan (BBJ) and the J-Kidney-Biobank (JKB). Additionally, we replicated our results in a meta-GWAS of BUN from the BBJ and the Taiwan Biobank (TWB). Finally, we explored the interactions between gut microbial taxa and serum proteins and evaluated the extent to which these proteins mediate the effects of the gut microbiota on CKD. The sources and detailed descriptions of all the data used in this study are provided in Supplementary Table S1.

The STROBE-MR guidelines were used to guide the design of this study [48], with the checklist available in Supplementary Table S.1.

### 2.2. Two-Sample MR Analysis of Gut Microbiota on CKD

We utilized summary data from a genome-wide association study (GWAS) involving 1539 individuals of Chinese descent, derived from the multi-omics 4D-SZ discovery cohort (Shenzhen, China), to obtain species-level information on the gut microbiota [49]. This dataset is notable for being the largest available database with species-level gut microbiota data in a Chinese population and offers a relatively homogeneous participant pool. The gut microbiota composition was characterized through whole-metagenomic sequencing of stool samples, which identified 500 unique microbial features (9 phyla, 3 classes, 14 orders, 32 families, 95 genera, 248 species, and 99 gut metabolic modules); gut metabolic modules (GMMs) reflect bacterial and archaeal metabolism specific to the human gut, with a focus on anaerobic fermentation processes (Supplementary Table S2). The assumptions for the instrumental variables and the potential influence of confounders in the association between gut microbiota and CKD are illustrated in Supplementary Figure S5.

We selected genetic variants of microbial taxa that met the GWAS significance threshold (*p* < 1 × 10^−5^), as defined in the original study, and had an effect allele frequency (EAF) > 0.01, following parameter settings referenced from previous studies [50]. While the threshold of *p* < 1 × 10^−5^ is less stringent than the conventional genome-wide significance threshold, it was chosen to increase the number of instrumental variables and the proportion of genetic variance explained by these predictors—a criterion commonly used in earlier MR studies on the gut microbiome. We then clumped the selected genetic variants to a linkage disequilibrium threshold of r^2^ < 0.1 within a ±1000-kilobase (kb) distance using the 1000 Genomes East Asian reference panel. Finally, we calculated the F-statistics for all SNPs for CKD-related microbiome features included in our analysis [51].

The strength of each instrumental variable was assessed using the F-statistic, calculated based on the proportion of variance in the exposure explained by the SNP (R^2^) and the sample size (N) using the following formula:

(1) $$F=\frac{\left(N-2\right)\cdot R^{2}}{\left(1-R^{2}\right)}$$

where R^2^ for each SNP was calculated using the following:

(2) $$R^{2}=\frac{2\cdot \beta^{2}\cdot EAF\cdot \left(1-EAF\right)}{2\cdot \beta^{2}\cdot EAF\cdot \left(1-EAF\right)+2\cdot N\cdot SE^{2}\cdot EAF\cdot \left(1-EAF\right)}$$

Here, β is the effect size of the SNP on the exposure, *SE* is the standard error, *EAF* is the effect allele frequency, and *N* is the sample size of the GWAS for the exposure.

For the primary analysis, we used summary statistics from the largest GWAS of CKD conducted in individuals of Chinese ancestry to date, comprising 489 cases and 75,531 controls from the China Kadoorie Biobank (CKB) [52]. The China Kadoorie Biobank (CKB), established between 2004 and 2008, is a large prospective cohort study involving over 512,000 adults aged 30–79 years from 10 diverse regions across China, aimed at investigating disease risk factors in the Chinese population. CKD cases were identified through multiple sources, including the parsing of free-text Chinese language disease descriptions and matching to a clinician-curated disease description library. These were then standardized into the International Classification of Diseases, 10th Revision (ICD-10), which codes incident disease events.

We replicated the results of the primary analysis in two independent datasets: Biobank Japan (BBJ) [53] (2117 cases and 174,345 controls) and J-Kidney-Biobank (JKB) [54] (382 cases of CKD in the J-Kidney-Biobank and 3471 controls participating in the TMM Comm-Cohort Study (JKB-ToMMo)). BBJ is a nationwide biobank in Japan that recruits participants based on the diagnosis of at least 1 of 47 target diseases [53]. The chronic kidney disease (CKD) data in the Biobank Japan (BBJ) study were derived from participants’ past medical history (PMH), standardized using the International Classification of Disease Controls (ICD-10) codes [53]. The J-Kidney-Biobank (JKB), established in 2020, aims to investigate the genetic and environmental factors influencing the progression of kidney diseases. All JKB participants were CKD patients, while the control population for the CKD GWAS was derived from the ToMMo cohort (Tohoku Medical Megabank Project Community-Based Cohort Study). Additionally, we validated the findings using chronic kidney disease traits, serum BUN levels, and estimated the glomerular filtration rate (eGFR) from meta-analyses of GWAS from Biobank Japan (BBJ) (n = 143,658) and the Taiwan Biobank (TWB) [55] (n = 101,294). BUN- and eGFR-related meta-analyses of GWASs involved the BBJ and TWB datasets (n = 241,112). The authors reported that the effect sizes of 2064 replicated eGFR-associated SNPs from 31 independent genomic risk loci were strongly and inversely correlated with eGFR and BUN (defined as kidney-relevant), which is consistent with the established understanding of kidney pathophysiology [55]. A summary of the CKD-related datasets used in our analyses is shown in Supplementary Table S1.

To estimate the causal effects, we primarily applied the inverse-variance weighted (IVW) method. This method yields efficient estimates when all genetic variants are valid instrumental variables.

To assess the robustness of our results and examine the potential violations of MR assumptions, we conducted multiple sensitivity analyses, including MR-Egger regression, the weighted median method, and MR-PRESSO (Pleiotropy RESidual Sum and Outlier). The MR-Egger regression detects and adjusts for directional horizontal pleiotropy, as indicated by a non-zero intercept. The weighted median method provides consistent estimates even when up to 50% of the instruments are invalid. MR-PRESSO identifies and corrects for horizontal pleiotropic outliers.

These sensitivity analyses were applied to all gut microbiota taxa with three or more instrumental variables. We also used Cochrane’s Q test to assess heterogeneity across SNPs. A two-sided *p*-value of less than 0.05 was considered statistically significant throughout.

### 2.3. Mediating Effects of Serum Protein on the Relationship Between the Gut Microbiota and CKD

To explore the potential causal pathways between gut microbial taxa and CKD, we performed mediation MR analyses focusing on CKD-related serum proteins. First, we performed two-sample MR analyses to explore the causal relationship between serum proteins, which come from GWAS data, for levels of 304 proteins in four sub-cohorts of the GNHS [56]. We next examined the relationships between significant gut microbial taxa and CKD-related serum proteins that were statistically significant in the MR analysis. Finally, we estimated the mediating effect of CKD-related serum proteins. The proportion mediated by the risk factors was calculated by dividing the indirect effect by the total effect [β1 × β2/β3] [57], where β1 represents the effect of the gut microbial feature on the risk factor, β2 represents the effect of the risk factor on CKD, and β3 represents the effect of the gut microbial taxon on CKD. Standard errors and *p*-values were obtained via the bootstrap method [57], and effect estimates were derived from two-sample MR analysis.

All the analyses were performed on the R platform (version 4.2.1). The “TwoSampleMR” and “Mendelian Randomization” packages were used for the statistical analyses.

## 3. Results

### 3.1. Instrument Variables

In this study, the number of instrumental variables (IVs) associated with each gut microbiota feature ranged from 3 to 40. SNPs were selected as IVs based on genome-wide significance (*p* < 1 × 10^−5^) from microbiome GWAS summary data. To ensure independence, linkage disequilibrium (LD) clumping was conducted using a threshold of r^2^ < 0.1 within a 1000 kb window. Palindromic SNPs with ambiguous strands were excluded. After harmonization with the CKD GWAS data, most SNPs were successfully matched, and proxy SNPs (r^2^ > 0.8) were used when necessary.

Notably, the F-statistics for all SNPs exceeded 10, with a median value above 20 for most traits, suggesting that weak instrument bias was unlikely. A comprehensive summary of IV characteristics is provided in Supplementary Table S3.

For key exposures, including Genus *Alistipes* and Species *Bifidobacterium catenulatum*–*Bifidobacterium pseudocatenulatum* complex—both of which have been previously implicated in kidney health—11 and 18 independent SNPs were retained, respectively, with median F-statistic values of 21.30 and 21.55. Detailed SNP-level information, including rsID, effect allele, β, SE, and F-statistic, is provided in Supplementary Tables S2 and S3.

### 3.2. MR Analysis of Gut Microbiota on CKD

**Figure 2:**
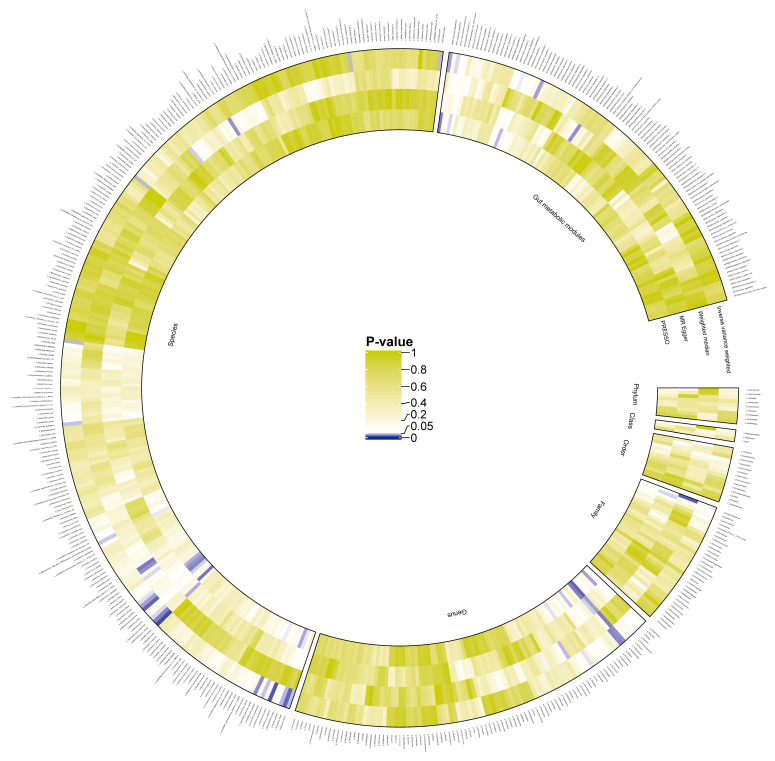
Preliminary MR estimates for gut microbiota and CKD risk. This figure presents the initial Mendelian randomization (MR) estimates assessing the relationship between gut microbiota and the risk of chronic kidney disease (CKD). The concentric circles represent estimates derived from different analytical methods in the following order, from the innermost to the outermost layer: PRESSO, MR-Egger, weighted median, and inverse variance weighted methods. The color intensity reflects statistical significance, with darker shades indicating smaller p-values.We identified 18 microbial features significantly associated with CKD, including one family, five genera, nine species, and three gut metabolic modules (GMMs) (Figure 3A). Specifically, the family *Moraxellaceae* (OR 0.87, 95% CI 0.76–1.00, *p* = 0.05), genus *Alistipes* (OR 1.20, 95% CI 1.03–1.40, *p* = 0.02), genus *Dickeya* (OR 1.22, 95% CI 1.02–1.46, *p* = 0.03), genus *Dorea* (OR 1.32, 95% CI 1.01–1.72, *p* = 0.04), genus *Klebsiella* (OR 1.16, 95% CI 1.01–1.33, *p* = 0.04), and genus *Parabacteroides* (OR 1.37, 95% CI 1.07–1.75, *p* = 0.01) were found to be significantly associated with CKD.

The initial analysis results examining the associations between gut bacterial taxa (instrumented by genetic variants) and CKD risk are illustrated in Figure 2 and summarized in Supplementary Table S4. All effect estimates are expressed as odds ratios (ORs) per one standard deviation (SD) increase in the respective exposure.

At the species level, *Bacteroides coprophilus* (OR 0.85, 95% CI 0.75–0.98, *p* = 0.03), *Bifidobacterium catenulatum*–*Bifidobacterium pseudocatenulatum* complex (OR 1.10, 95% CI 1.00–1.21, *p* = 0.04), *Bifidobacterium longum* (OR 1.23, 95% CI 1.02–1.48, *p* = 0.03), *Gemella sanguinis* (OR 1.20, 95% CI 1.00–1.43, *p* = 0.04), *Mobiluncus curtisii* (OR 0.88, 95% CI 0.78–0.99, *p* = 0.04), *Peptoniphilus duerdenii* (OR 1.14, 95% CI 1.01–1.28, *p* = 0.03), *Peptostreptococcus stomatis* (OR 1.15, 95% CI 1.02–1.29, *p* = 0.02), *Porphyromonas gingivalis* (OR 1.18, 95% CI 1.00–1.39, *p* = 0.05), and *Streptococcus pneumoniae* (OR 1.29, 95% CI 1.04–1.59, *p* = 0.02) were significantly linked with CKD.

Furthermore, three microbial functional modules were found to have a causal association with CKD: *MF0031 glutamate degradation II* (OR 1.28, 95% CI 1.04–1.59, *p* = 0.02), *MF0047 glutamine degradation II* (OR 0.35, 95% CI 0.13–0.95, *p* = 0.04), and *MF0102 sulfate reduction (dissimilatory)* (OR 0.78, 95% CI 0.62–0.98, *p* = 0.03).

The causal relationship between the genus *Parabacteroides* and CKD was further supported by multiple MR sensitivity analyses, including the MR-Egger, weighted median, and MR-PRESSO approaches. The MR-Egger regression showed no evidence of directional pleiotropy (intercept *p* = 0.111), and Cochrane’s Q statistic did not indicate heterogeneity across instruments (all *p* > 0.05). MR-PRESSO did not identify any significant outliers that could bias the estimates.

Similarly, for other taxa with suggestive or nominal associations (e.g., *Alistipes*, *Bifidobacterium catenulatum*–*pseudocatenulatum* complex), the estimates remained directionally consistent across sensitivity methods, with no evidence of pleiotropy or outlier influence. Detailed results for all taxa are summarized in Supplementary Tables S5–S7.

**Figure 3:**
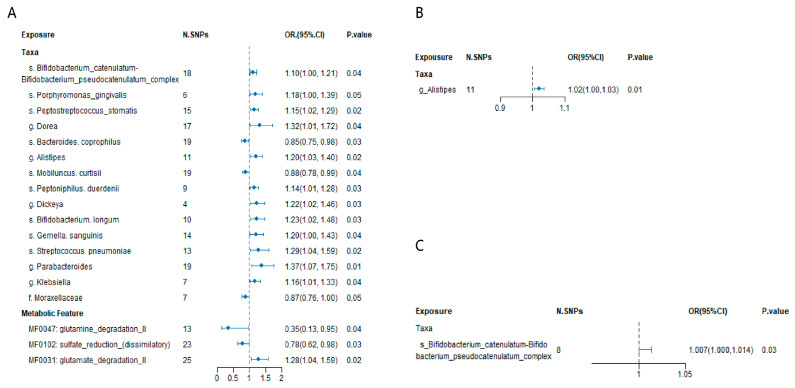
(**A**) Mendelian randomization analysis of significant gut microbial taxa and chronic kidney disease in the Chinese population. SNP: single nucleotide polymorphism. N. SNPs: Number of SNPs used for the estimation of the causal effects. (**B**) Replication of gut microbial taxa and chronic kidney disease using GWAS data from J-Kidney-Biobank. (**C**) Replication of gut microbial taxa and chronic kidney disease using blood urea nitrogen (BUN) GWAS data from meta data of TW and BBJ. Odds ratios (ORs), 95% confidence interval (95% CI), and *p*-values were calculated using the inverse variance weighted method. The prefixes “g.”, “s.”, “f.”, and “MF” in the taxa column represent genus, species, family, and the metabolic feature (gut microbiome modules, GMMs), respectively. The size of blue dot represents the stand error of the Odds ratios.

In the validation analysis using data from the J-Kidney-Biobank (JKB), the genus *Alistipes* was significantly associated with CKD, with an odds ratio (OR) of 1.02 and a 95% confidence interval (CI) of 1.00–1.03 (*p* = 0.01) (Figure 3B, Supplementary Table S8). No gut microbiota was replicated with chronic renal failure from the BBJ (Supplementary Table S9). Additionally, the species *Bifidobacterium catenulatum*–*Bifidobacterium pseudocatenulatum* complex was further validated via a Mendelian randomization analysis, with blood urea nitrogen (BUN) levels as the outcome of a meta-analysis of TW and BBJ. The association yielded an OR of 1.007 with a 95% CI of 1.000–1.014 (*p* = 0.03) (Figure 3C, Supplementary Table S9). No gut microbiota was replicated with the estimated glomerular filtration rate (eGFR) levels as the outcome of a meta-analysis of the TW and BBJ (Supplementary Table S10).

### 3.3. Mediating Effect of CKD-Related Serum Protein

A total of 304 circulating serum proteins were measured in plasma samples from the Guangzhou Nutrition and Health Study (GNHS). Among these, 17 CKD-related serum proteins were selected for mediation analysis based on their known biological relevance to kidney disease and previous associations with gut microbiota. The selected proteins were subject to quality control measures [1], and those with nominally significant associations with both gut microbiota and CKD outcomes were identified as potential mediators.

**Figure 4:**
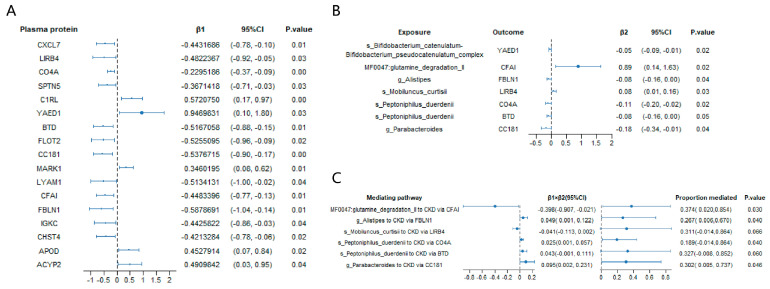
Mediation MR analysis of the causal effect of gut microbiota on chronic kidney disease via serum proteins. (**A**) Mendelian randomization (MR) analysis of serum protein on chronic kidney disease; (**B**) MR analysis of significant gut microbiota taxa on significant CKD-related serum proteins; (**C**) estimates of the effect of gut microbiota on chronic kidney disease explained by risk factors. β1, the effect of the serum protein on chronic kidney disease. β2, the effect of the gut microbial taxon on the serum protein. *p*-values were calculated from the inverse variance weighted method. The prefixes “g.”, “s.”, and “MF” in the taxa column represent genus, species, and the metabolic feature (gut microbiome modules, GMMs), respectively.

As a result, 17 serum proteins, including CXCL7, LIRB4, CO4A, SPTN5, C1RL, YAED1, BTD, FLOT2, CC181, MARK1, LYAM1, CFAI, FBLN1, IGKC, CHST4, APOD, and ACYP2, that were significantly associated with CKD according to the MR analysis (Figure 4A and Supplementary Table S11).

In the MR analysis exploring the associations between gut microbial taxa and these 17 significant serum proteins, we found that the genus *Alistipes* was significantly associated with FBLN1 (β = −0.08, 95% CI = −0.16 to 0.00, *p* = 0.04). Similarly, the genus *Parabacteroides* was significantly associated with CC181 (β = −0.18, 95% CI = −0.34 to −0.01, *p* = 0.04). At the species level, *Peptoniphilus duerdenii* was significantly associated with both CO4A (β = −0.11, 95% CI = −0.20 to −0.02, *p* = 0.02) and BTD (β = −0.08, 95% CI = −0.16 to 0.00, *p* = 0.05), whereas *Mobiluncus curtisii* was significantly associated with LIRB4 (β = 0.08, 95% CI = 0.01 to 0.16, *p* = 0.03). The species *Bifidobacterium catenulatum*–*Bifidobacterium pseudocatenulatum* complex was significantly associated with YAED1 (β = −0.05, 95% CI = −0.09 to −0.01, *p* = 0.02). Additionally, the gut metabolic module (GMM) *MF0047: glutamine degradation II* was significantly associated with CFAI (β = 0.89, 95% CI = 0.14 to 1.63, *p* = 0.02) (Figure 4B and Supplementary Table S12).

Furthermore, the mediation analysis revealed that the proportion of the mediation effect of genus *Alistipes* on CKD risk through the protein FBLN1 was 26.70% (95% CI = 0.006 to 0.6700, *p* = 0.04) (Figure 4C). The mediation effect of the genus *Parabacteroides* on CKD risk through protein CC181 was 30.20% (95% CI = 0.005 to 0.737, *p* = 0.05). Similarly, species *Peptoniphilus duerdenii* had a mediation effect on CKD risk through protein CO4A of 18.90% (95% CI = 0.005 to 0.737, *p* = 0.05). Finally, the mediating effect of the gut metabolic module (GMM) *glutamine degradation II* on CKD risk via protein CFAI was 37.40% (95% CI = 0.020 to 0.854, *p* = 0.03) (Figure 4C).

## 4. Discussions

**Figure 5:**
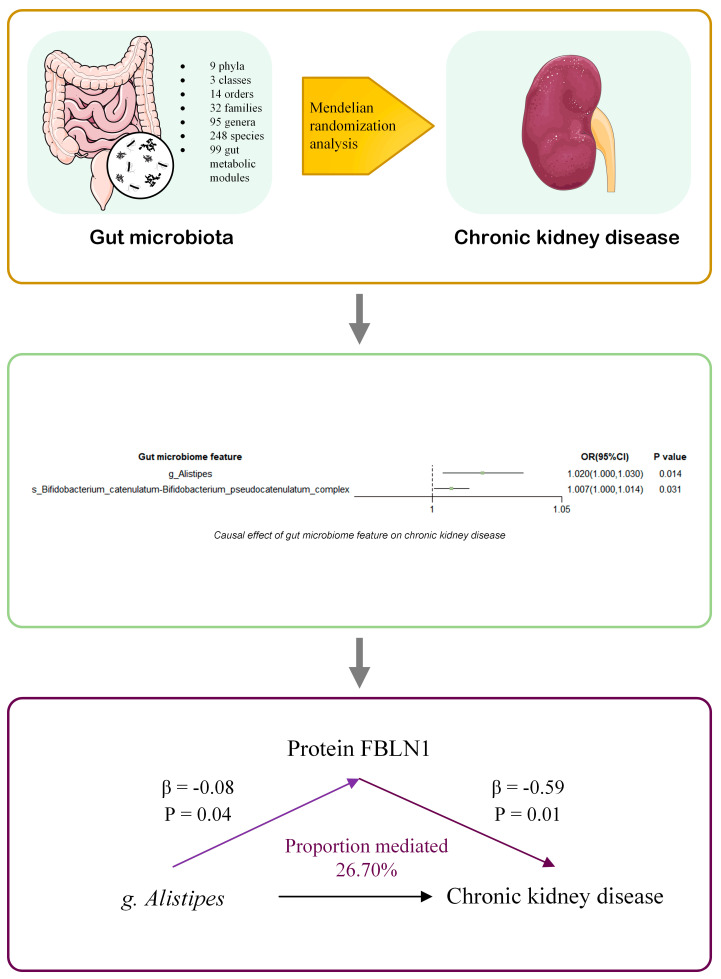
Summary of the study. We conducted a two-sample Mendelian randomization (MR) analysis using summary statistics from genome-wide association studies (GWAS) of 500 gut microbial traits (4D-SZ) and large-scale GWAS of chronic kidney disease (CKD). We identified two microbial taxa causally associated with CKD: the *Bifidobacterium catenulatum*–*Bifidobacterium pseudocatenulatum* complex and the genus *Alistipes*. Mediation MR analyses were performed for plasma protein levels. Our findings suggest that the protein FBLN1 may mediate the effect of *Alistipes* on CKD. OR: odds ratio. The prefixes “g.” and “s.” refer to genus and species, respectively.

In this comprehensive Mendelian randomization (MR) study, we identified a gut microbial taxon, the genus *Alistipes*, which was significantly associated with the risk of CKD in both the discovery and validation cohorts, as summarized in Figure 5. To the best of our knowledge, this study provides novel insights into the potential connection between this specific gut microbial feature and CKD in the Chinese population. Moreover, our findings indicate that the serum protein FBLN1 may mediate the influence of the genus *Alistipes* on CKD risk. These results offer MR-based evidence for a potential causal link between certain gut microbiota features and CKD and suggest a possible mediation pathway involving the gut microbiota, serum proteins, and CKD progression.

The genus *Alistipes* comprises Gram-negative anaerobic bacteria that are resistant to bile and a variety of antibiotics. They have the ability to convert lactic acid into acetic acid, propionic acid, and other short-chain fatty acids (SCFAs). SCFAs, including butyrate, propionate, and acetate, are microbial metabolites whose availability is influenced by environmental factors such as diet and antibiotic use [58]. SCFAs are known to regulate epithelial barrier function, modulate immune responses, and exert anti-inflammatory roles and affect immunity at extra-intestinal sites, including the liver, lungs, and brain [58]. Although the genus *Alistipes* produces SCFAs with potential benefic function, observational studies suggest that it may also play a pathogenic role in several conditions, such as colorectal cancer, hypertension, and mental health disorders (e.g., anxiety and depression). The role of *Alistipes* appears to be context-dependent, exhibiting both protective and harmful effects depending on the disease type and host environment. Multiple observational studies have demonstrated a relationship between *Alistipes* and the development and progression of chronic kidney disease (CKD). A study from China revealed that increases in *Alistipes* (OR = 1.037; 95% CI 1.007, 1.068) and Bifidobacterium were linked to an increased incidence of CKD [59]. Similarly, research in Korea reported that the abundance of *Alistipes* and *Oscillibacter* increased with CKD severity and was positively correlated with the serum levels of p-cresyl glucuronide and indoxyl sulfate [60]. A Taiwanese study revealed that the abundance of *Alistipes* was significantly correlated with the total forms of indoxyl sulfate (IS) and p-cresyl sulfate (pCS) [61]. Moreover, another Chinese study revealed that *Alistipes* (primarily *A. finegoldii* and *A. shahii*) was highly enriched in end-stage renal disease (ESRD) patients, with *A. shahii* being an indole-positive bacterium. Additionally, *A. shahii* was found to contribute to increased levels of uremic toxins, including AAA degradation products, secondary bile acids (SBAs), and trimethylamine-N-oxide (TMAO) [17]. Our mediation analysis revealed that FBLN1 levels may mediate the association between genus *Alistipes* and CKD. This is the first study to report a significant association between the species genus *Alistipes*, FBLN1, and CKD risk. Previous studies have shown that elevated serum levels of fibulin-1 are associated with diabetes and impaired kidney function [46], as well as with hemodynamic cardiovascular risk markers [46]. Other studies have indicated that fibulin-1 plays a role in cardiac and vascular remodeling in chronic kidney disease [47,62]. Furthermore, fibulin-1 may serve as a potential therapeutic target in diabetic nephropathy [63]. Collectively, our findings suggest a potential causal pathway linking gut microbiota, serum proteins, and CKD progression, thereby providing novel insights into CKD pathogenesis.

Although *Bifidobacterium* has been used as a probiotic in the treatment of chronic kidney disease (CKD) with positive outcomes—such as a significant reduction in total serum p-cresol levels in CKD patients after treatment with symbiotic agents [64]—the protective effects of *Bifidobacterium* on CKD remain uncertain. An observational study reported that *Bifidobacterium* was associated with CKD incidence (OR = 1.034; 95% CI: 1.003–1.065) [59]. Our findings indicate that the *Bifidobacterium catenulatum*–*Bifidobacterium pseudocatenulatum* complex is positively associated with CKD prevalence. According to the List of Prokaryotic Names with Standing in Nomenclature (LPSN) [65], there are approximately 84 species within the genus *Bifidobacterium*. As the association of the species *Bifidobacterium catenulatum*–*Bifidobacterium pseudocatenulatum* complex with CKD has not been previously reported, further studies are warranted to elucidate this relationship.

While our findings suggest a statistically significant association between the *Bifidobacterium catenulatum*–*Bifidobacterium pseudocatenulatum* complex and CKD risk, we emphasize that this does not imply a direct pathogenic effect of these taxa. It is possible that the observed association reflects shared genetic determinants influencing both host traits and microbial composition or unmeasured environmental or metabolic factors. Given the complexity of host–microbiota interactions, this finding should be interpreted with caution. We consider this result to be hypothesis-generating and not confirmatory of causality. Further mechanistic studies and replication in independent cohorts are needed to clarify the nature and direction of this association.

Overall, although some studies suggest a potential link between genus *Alistipes* and the *Bifidobacterium catenulatum*–*Bifidobacterium pseudocatenulatum* complex with CKD, the existing evidence is limited and of relatively low quality. Therefore, future research should include larger clinical trials, as well as animal and cellular mechanistic studies, to better understand the health effects and underlying mechanisms of these bacteria. Moreover, given the heterogeneity of gut microbiota across different populations, especially considering the genetic differences between East Asian populations and other ethnic groups, it is important to further validate these findings in more diverse cohorts. This will help improve the generalizability of the results and provide a more comprehensive understanding of the microbiota–CKD relationship.

Although all GWAS datasets used in this study, including the 4D-SZ cohort, the China Kadoorie Biobank (CKB), Biobank Japan (BBJ), and the J-Kidney-Biobank (JKB), applied standard quality control procedures and adjusted for population stratification using principal components analysis (PCA), some degree of genetic heterogeneity may still exist between different East Asian subpopulations. Differences in environmental exposures, cultural factors, and genetic architecture between Chinese and Japanese populations could potentially introduce heterogeneity in effect estimates. However, the overall genetic background across East Asian populations is relatively homogeneous compared to multi-ethnic designs, which may mitigate substantial bias. Moreover, the consistent replication of associations across independent cohorts in our study enhances the robustness of our findings. Nonetheless, we acknowledge this as a limitation, and future studies using ancestry-matched cohorts or meta-analytic approaches should further validate these associations.

Previous Mendelian randomization (MR) studies have investigated the relationship between the gut microbiota and CKD [37,39,40,41]. The gut microbiota data from the MiBioGen consortium included 18,340 multiancestry participants and 211 gut microbiota taxa characterized at the genus level. Our study yielded results that were not entirely consistent with those of previous studies, which may be attributed to the heterogeneity of the population or the effect of heterogeneity within the subclassification of specific taxa. Interestingly, in our findings, the genus *Streptococcus*, particularly the species *Streptococcus pneumoniae*, was associated with a reduced incidence of CKD, a result that appears inconsistent with the prior literature. This discrepancy may be due to the unique role of this specific species. Clinically, *Streptococcus pneumoniae* is the most frequently identified pathogen, and patients with *Streptococcus pneumoniae* infections combined with CKD exhibit higher mortality rates [66]. Moreover, infections with *Streptococcus pneumoniae* in adults have been linked to an increased incidence of ESRD [67]. Therefore, we speculate that ectopic colonization of *Streptococcus pneumoniae* in the gut may contribute to an elevated risk of CKD.

Moreover, we acknowledge the well-established evidence that gut microbiota alterations are often a consequence of CKD progression and its associated metabolic and immune disturbances. Recent reviews [68] emphasize that dysbiosis is frequently observed in CKD patients, suggesting that a reverse causal direction may exist.

Ideally, a bidirectional Mendelian Randomization (MR) analysis could be conducted to explore the possibility of reverse causation from CKD to gut microbiota. However, due to the current limitations of available GWAS data—particularly the fact that the gut microbiota GWAS we used (4D-SZ cohort) are only accessible as summary statistics for use as exposures—it is not feasible to use microbiota as outcomes in standard two-sample MR analyses.

To further ensure the robustness of our MR findings, we performed a series of sensitivity analyses, including MR-Egger regression, weighted median estimation, and MR-PRESSO. For key associations such as the genus *Alistipes* and CKD, the results remained directionally consistent across these methods. No evidence of directional pleiotropy was observed (MR-Egger intercept *p* > 0.05), and Cochran’s Q tests suggested no substantial heterogeneity. Additionally, the MR-PRESSO global test and outlier test did not detect significant pleiotropic outliers. These findings collectively support the validity of our causal inference and reduce the likelihood that the observed associations are driven by horizontal pleiotropy or invalid instruments. Despite this constraint, we have included a discussion of the potential for reverse or bidirectional relationships, and we interpret our findings with appropriate caution. While our study suggests a potential causal role of specific gut microbial taxa in CKD development, this does not preclude the possibility that CKD itself may also influence the gut microbiota.

Our study possesses several notable strengths. First, we conducted a Mendelian randomization (MR) analysis utilizing the largest Chinese population-based GWAS dataset for CKD. Second, our findings were validated via independent GWAS data from a separate population. Finally, we performed mediation analysis to explore the potential mechanistic pathways linking the gut microbiota to CKD.

## 5. Limitations

This study has several limitations that should be acknowledged. First, while the GWAS summary data for species-level gut microbiome taxa in the Chinese population constitute the largest dataset available to date, it may still lack the statistical power to detect all potential causal relationships owing to the high heterogeneity of the gut microbiota across populations. A *p*-value threshold of *p* < 1 × 10^−5^ was used as the genome-wide significance level to define significant genetic loci, which is consistent with the approach used in primary GWASs and other MR studies of gut microbiota. Despite this, the instrumental variables used in our analysis were strong and suitable for downstream analyses. However, given the high variability of gut microbiota and the relatively small proportion of variance explained by host genetics, caution is warranted when interpreting the inferred causal relationships. Second, previous studies have reported differences in gut dysbiosis associated with various etiologies of CKD. However, owing to the absence of detailed phenotyping for CKD in the original GWAS dataset, we were unable to explore the causal relationships between the gut microbiota and specific CKD etiologies. Third, while we applied several methods to assess and adjust for potential heterogeneity or pleiotropic effects, the influence of unknown sources of heterogeneity or pleiotropy cannot be entirely excluded. Although microbiome GWAS was adjusted for some known confounders (e.g., BMI, diet), unmeasured confounders may still remain which have not been fully accounted for and could impact the validity of the results. Fourth, we did not perform mode-based estimation for all exposure–outcome pairs because several taxa had only a small number of valid instrumental variables (as few as three), making the application of mode-based methods unreliable. Importantly, these taxa were not the focus of our main findings. Finally, as our analysis was primarily conducted in a Chinese population, caution should be exercised when generalizing the findings to other ethnic groups, as there may be ethnicity-specific associations between host genomes and the gut microbiota.

## 6. Conclusions

In this Mendelian randomization study, we identified 18 gut microbial features with potential causal effects on CKD, of which two microbial taxa were validated in independent datasets. These findings provide genetic evidence that alterations in the gut microbiota may serve as significant predisposing factors for CKD development. These results offer novel insights into the pathophysiology of CKD and highlight potential therapeutic targets for its prevention and management. Further studies are warranted to replicate these findings and to elucidate the underlying mechanisms involved.

## Data Availability

GWAS summary data for gut microbiota from the 4D-SZ cohort (from Shenzhen, China) discovery cohort are available at https://ftp.cngb.org/pub/CNSA/data2/CNP0000794/microbiome/ (access date: 15 November 2024). Chronic kidney disease from the China Kadoorie Biobank (CKB) by Walters et al. at https://pheweb.ckbiobank.org/pheno/n18 (access date: 15 November 2024). Chronic renal failure from the Biobank Japan by Sakaue S. et al. at https://pheweb.jp/pheno/Chronic_Renal_Failure (access date: 15 November 2024). Chronic kidney disease from the J-Kidney-Biobank (JKB) dataset by Sugawara, Y. et al. at https://jmorp.megabank.tohoku.ac.jp/gwas-studies/TGA000006 (access date: 15 November 2024). Meta-GWAS of BUN and eGFR from the discovery dataset of Biobank Japan (BBJ) and Taiwan Biobank (TWB) by Chen, HL. et al. at https://doi.org/10.6084/m9.figshare.24356587 (access date: 15 November 2024). Plasma proteins level from the Guangzhou Nutrition and Health Study (GNHS) by https://omics.lab.westlake.edu.cn/data/proteins/ (access date: 15 November 2024).

## Supplementary Materials

The following supporting information can be downloaded at: https://www.mdpi.com/article/10.3390/biomedicines13061397/s1, Supplementary Figure S1. Scatter plots of the MR analyses for the association of 18 gut bacterial taxa and the risk of chronic kidney disease. Supplementary Figure S2. Funnel plots from 18 gut bacterial taxa on the risk of CKD. Supplemental Figure S3. Leave-one-out analyses for the causal estimates of 18 gut bacterial taxa on CKD. Supplementary Figure S4. Two-sample mediation Mendelian randomization analysis of gut microbiota on CKD via plasma proteins (Two step MR method). Supplementary Figure S5. Flowchart of the Mendelian Randomization (MR) analysis for assessing the causal relationship between gut microbiota modules and chronic kidney disease (CKD). Supplementary Table S.1. STROBE-MR checklist. Supplementary Table S1. Two-sample Mendelian Randomization study data source. Supplementary Table S2. 500 gut microbiome features enrolled in this study. Supplementary Table S3. Instruments variables information for the 500 gut microbiome features. Supplementary Table S4. MR estimates from gut microbiota on CKD risk (under the filter conditions of linkage disequilibrium r2 < 0.1 and genetic distance of 1000 kb). Supplementary Table S5. The MR Pleiotropy Residual Sum and Outlier (MR-PRESSO) results of all the gut microbiota on CKD. Supplementary Table S6. Cochrane’s Q and Egger Intercept Results. Supplementary Table S7. Sensitivity MR analysis of significant gut microbiota taxa on CKD. Supplementary Table S8. MR estimates from gut microbiota on CKD risk using JMOP dataset. Supplementary Table S9. MR estimates from gut microbiota on CKD risk using BBJ dataset. Supplementary Table S10. MR estimates from gut microbiota on CKD risk using meta-GWAS of eGFR dataset and MR estimates from gut microbiota on CKD risk using meta-GWAS of BUN dataset. Supplementary Table S11. Causal effect estimates of plasma protein on CKD. Supplementary Table S12. Causal effect estimates of gut microbiota on plasma protein.
