# Supplementary material for: Association Between Gut Microbiota and Chronic Kidney Disease: A Two-Sample Mendelian Randomization Study in a Chinese Population
Source: Biomedicines. 2025 Jun 6;13(6):1397. doi: 10.3390/biomedicines13061397 (PMC12190567; doi:10.3390/biomedicines13061397)
Supplement: Supplementary file 1 [file biomedicines-13-01397-s001.zip › supplemental-materials-reviesed.pdf]

Supplementary Material

Title Causal Association Between Gut Microbiota and Chronic Kidney Disease: A Two-Sample Mendelian Randomization Study in a Chinese Population

Authors

Wenjian Lin†, Zixin Liang†, Junxuan Fang, Yu Liu, Lei Lei, Jiawen Lin, Bin Xia, Zhihua Zheng , Jingqiu Yuan*, Chun Tang*

Supplementary Figure

Supplementary Figure S1. Scatter plots of the MR analyses for the association of 18 gut bacterial taxa and the risk of chronic kidney disease. (**Page 9 in main text**)

Supplementary Figure S2. Funnel plots from 18 gut bacterial taxa on the risk of CKD. (**Page 10 in main text**)

Supplemental Figure S3. Leave-one-out analyses for the causal estimates of 18 gut bacterial taxa on CKD (**Page 11 in main text**)

Supplementary Figure S4. Two-sample mediation Mendelian randomization analysis of gut microbiota on CKD via plasma proteins (Two step MR method).
(Page 14 in main text).

Supplementary Figure S5. Flowchart of the Mendelian Randomization (MR) analysis for assessing the causal relationship between gut microbiota modules and chronic kidney disease (CKD). (**Page 5 in main text)**.

Supplementary Tables

Supplementary Table S.1. STROBE-MR checklist (**Page 13 in main text**)

Supplementary Figure S1. Scatter plots of the MR analyses for the association of 18 gut bacterial taxa and the risk of chronic kidney disease.

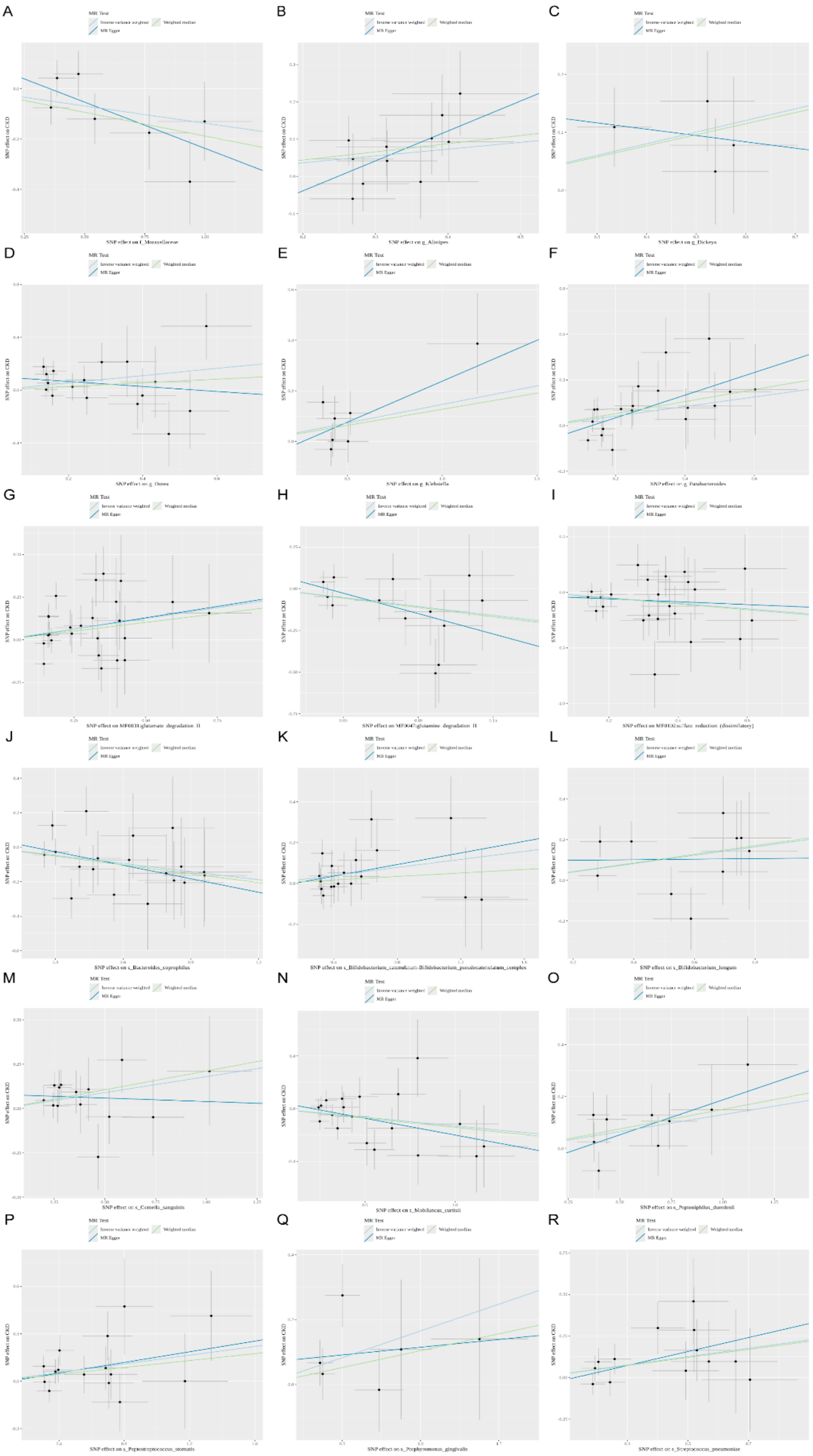

Supplementary Figure S2. Funnel plots from 18 gut bacterial taxa on the risk of CKD.

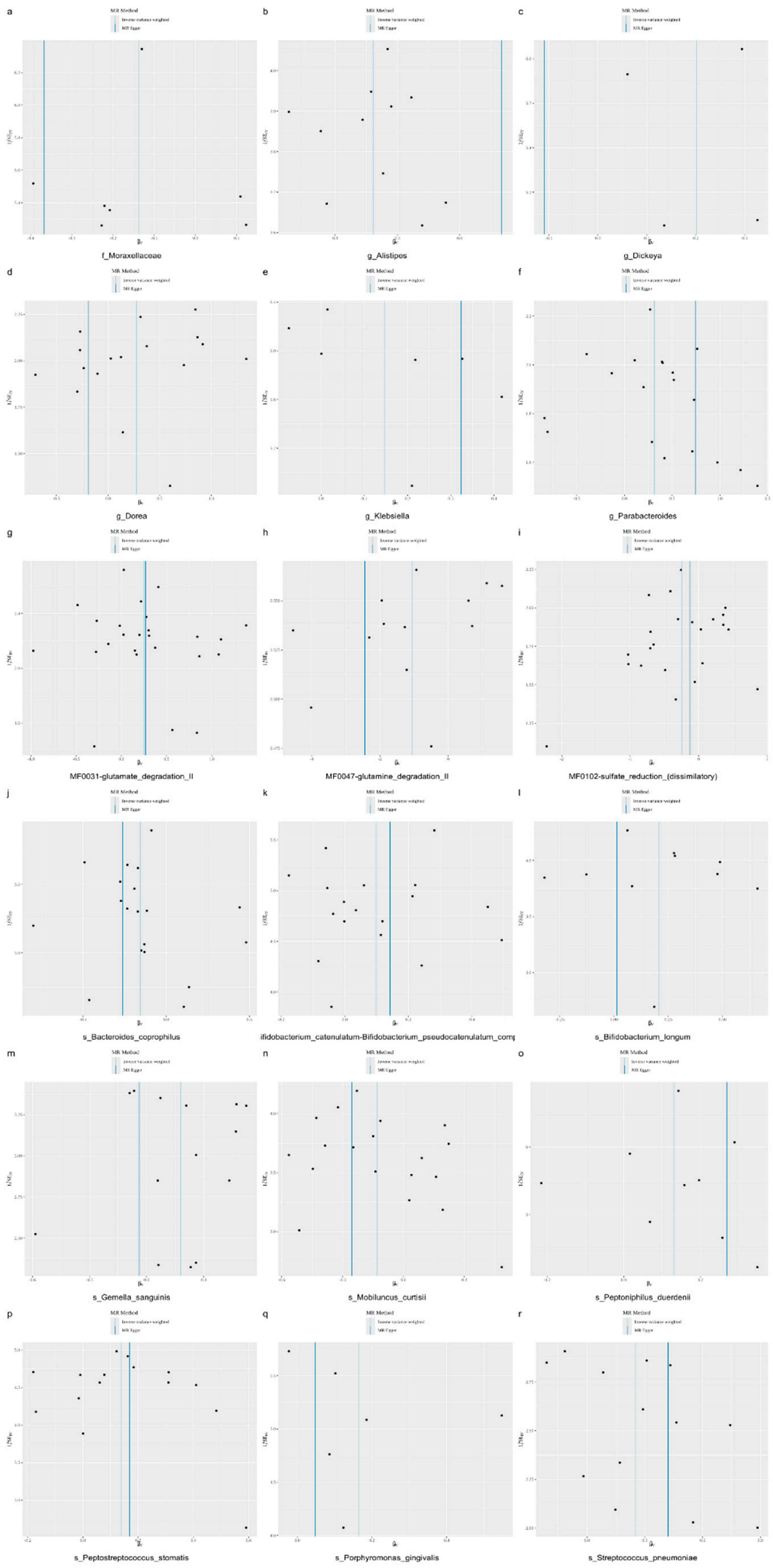

Supplementary Figure S3. Leave-one-out analyses for the causal estimates of 18 gut bacterial taxa on CKD

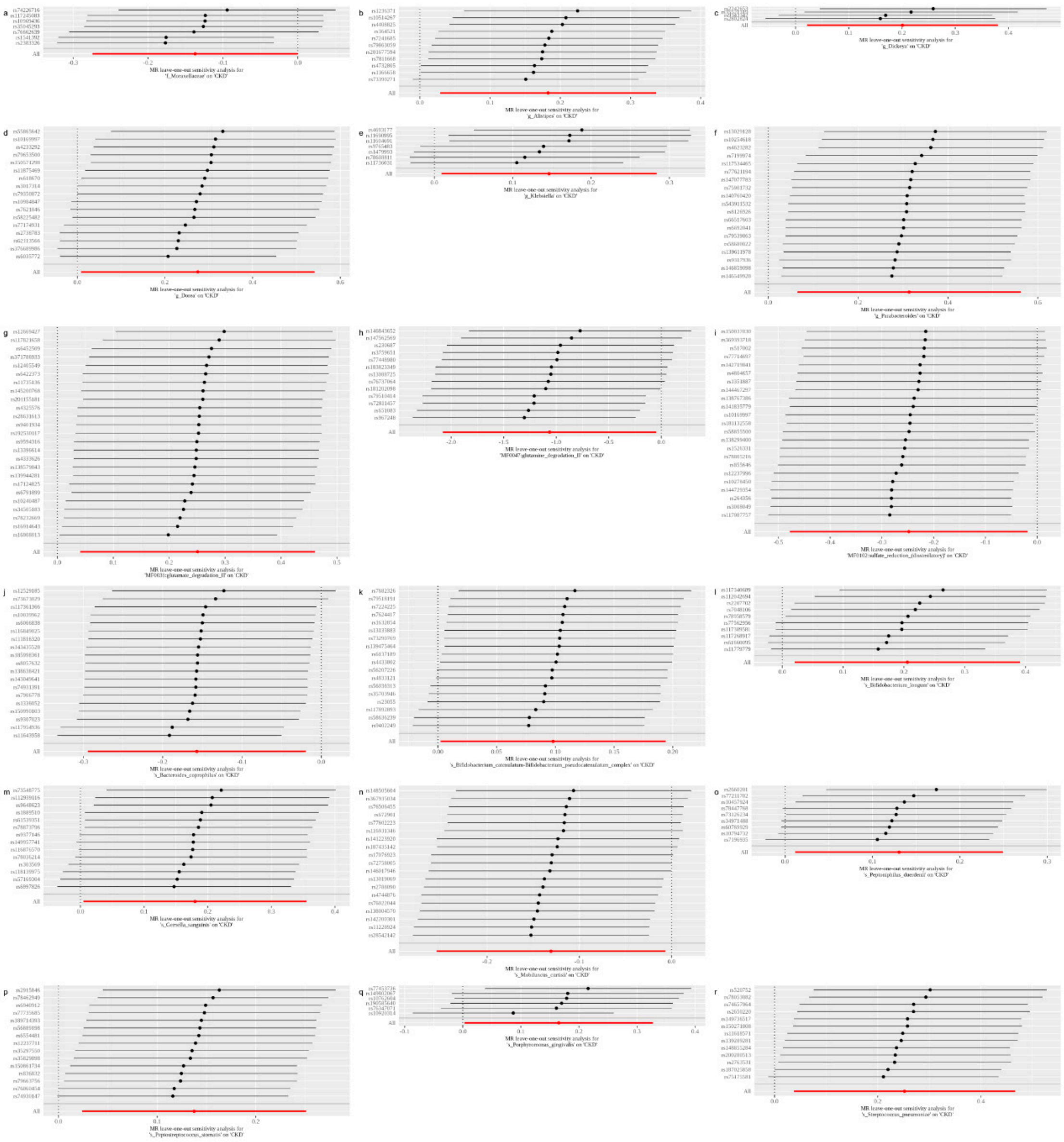

Supplemental Figure S4. Two-sample mediation Mendelian randomization analysis of gut microbiota on CKD via plasma proteins (Two step MR method).

Step 1. Causal effect estimation of plasma protein on Chronic kidney disease.

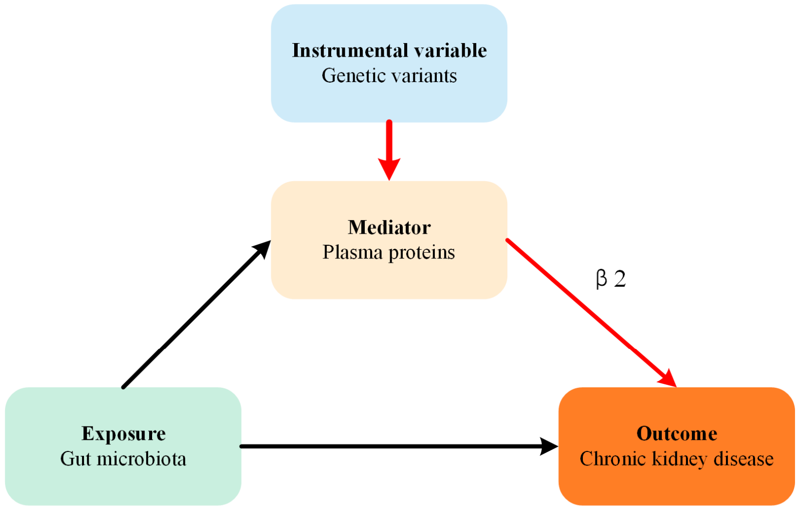

Step 2. Causal effect estimation of gut microbiome on plasma protein.

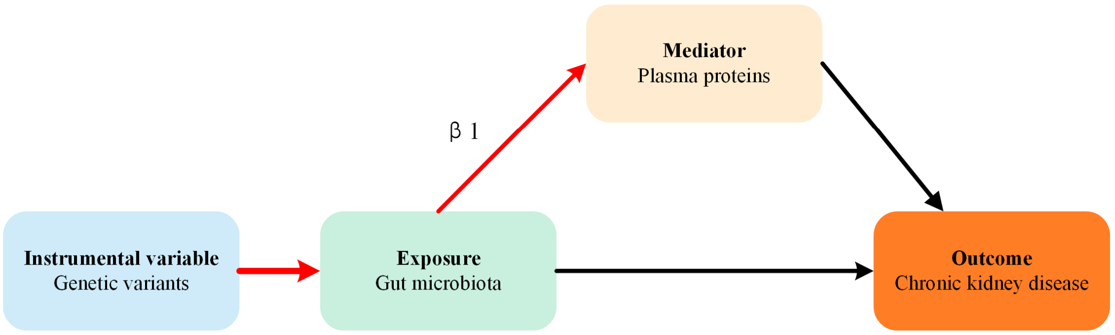

Supplemental Figure S5. Flowchart of the Mendelian Randomization (MR) analysis for assessing the causal relationship between gut microbiota modules and chronic kidney disease (CKD).

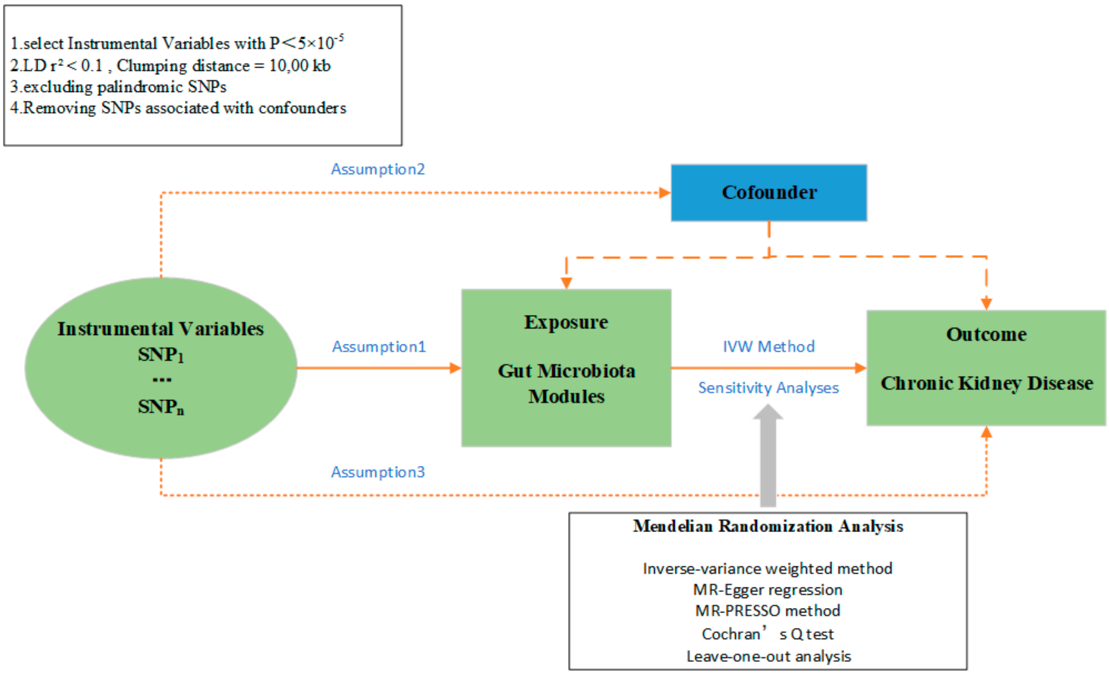

Supplementary Table S.1. STROBE-MR checklist

Item	Complete/location
**1. Title and Abstract:** "Mendelian randomization" is named both in the title and the abstract	Complete
**Introduction**	
**2. Background:** Explain the scientific background and rationale for the reported study. Is causality between exposure and outcome plausible? Justify why MR is a helpful method to address the study question.	Concept of Mendelian randomization and specific request for Mendelian randomization were explained in the third paragraph of the introduction (**Page 2**).
**3. Objectives:** State specific objectives clearly, including pre-specified causal hypotheses (if any).	The causal question has been stated in the fourth paragraph of the introduction (**Page 2**).
**Methods**	
**4. Study design and data sources:** Present key elements of study design early in the paper.<br>Consider including a table listing sources of data for all phases of the study. For each data source contributing to the analysis, describe the following:<br>a) Describe the study design and the underlying population from which it was drawn. Describe also the setting, locations, and relevant dates, including periods of recruitment, exposure, follow-up, and data collection, if available.<br>b) Give the eligibility criteria, and the sources and methods of selection of participants.<br>c) Explain how the analyzed sample size was arrived at.<br>d) Describe measurement, quality and selection of genetic variants.<br>e) For each exposure, outcome and other relevant variables, describe methods of assessment and, in the case of diseases, the diagnostic criteria used.<br>f) Provide details of ethics committee approval and participant informed consent, if relevant.	All necessary information about the GWAS studies been used in this study have been described in the method section.<br>The genetic predictor selection process has been described in in the Methods section "Two sample MR analysis of gut microbiota on CKD" (**Page 4**).<br>Ethics approval and informed consent info in the method section (**Page 17**).

**5. Assumptions:** Explicitly state assumptions for the main analysis (e.g. relevance, exclusion, independence, homogeneity) as well assumptions for any additional or sensitivity analysis.	The relevance assumption was validated using the strength of the genetic predictors of each gut microbiota feature estimated by F statistics (**Page 4**).<br>The exclusion restriction assumption was tested using the following sensitivity approaches: MR Egger regression; weighted median analysis; and MR-PRESSO (**Page 5**).
**6. Statistical methods main analysis**<br>Describe statistical methods and statistics used.<br>a) Describe how quantitative variables were handled in the analyses (i.e., scale, units, model).<br>b) Describe the process for identifying genetic variants and weights to be included in the analyses (i.e, independence and model). Consider a flow diagram.<br>c) Describe the MR estimator, e.g. two-stage least squares, Wald ratio, and related statistics. Detail the included covariates and, in case of two-sample MR, whether the same covariate set was used for adjustment in the two samples.<br>d) Explain how missing data were addressed.<br>e) If applicable, say how multiple testing was dealt with.	All necessary information is described in the methods section “Two sample MR analysis of gut microbiota on CKD” (**Page 4** and **Page 5**).
**7. Assessment of assumptions: Describe any methods used to assess the assumptions or justify their validity.**	The relevance assumption was validated using the strength of the genetic predictors of each gut microbiota taxa estimated by F statistics (**Page 4**).<br>The exclusion restriction assumption was tested using the following sensitivity approaches: MR Egger regression; weighted median analysis; and MR-PRESSO (**Page 5**).
**8. Sensitivity analyses:** Describe any sensitivity analyses or additional analyses performed.	The Mendelian randomization sensitivity analyses and additional analyses have been described in "Two sample MR analysis of gut microbiota on CKD" section of the Methods (**Page 4** and **Page 5**).

**9. Software and pre-registration**<br>a) Name statistical software and package(s), including version and settings used.<br>b) State whether the study protocol and details were pre-registered (as well as when and where).	a) All statistical software and settings used are described in the method section (**Page 6**).<br>b) The analysis plan was described in the " Study design" section of the Methods (**Page 3**).
**Results**	
**10. Descriptive data**<br>a) Report the numbers of individuals at each stage of included studies and reasons for exclusion. Consider use of a flow-diagram.<br>b) Report summary statistics for phenotypic exposure(s), outcome(s) and other relevant variables<br>(e.g. means, standard deviations, proportions).<br>c) If the data sources include meta-analyses of previous studies, provide the number of studies, their reported ancestry, if available, and assessments of heterogeneity across these studies. Consider using a supplementary table for each data source.<br>d) For two-sample Mendelian randomization:<br>i. Provide information on the similarity of the genetic variant-exposure associations between the exposure and outcome samples.<br>ii. Provide information on extent of sample overlap between the exposure and outcome data sources.	a) Information is given in the " Study design" section of the Methods (**Page 3**).<br>b) We described the detailed information of the summary statistics for our analysis in Methods (**Page 4 and Page 5**).<br>c) We give this information in **Figure 1**.<br>d) We provide this information in the "Study design" section of the Methods (**Page 4**).
**11. Main results**<br>a) Report the associations between genetic variant and exposure, and between genetic variant and outcome, preferably on an interpretable (e.g. comparing 25th and 75th percentile of allele count or genetic risk score, if individual-level data available).<br>b) Report causal effect estimate between exposure and outcome, and the measures of uncertainty from the MR analysis. Use an intuitive scale, such as odds ratio, or relative risk, per standard deviation difference.	a) Genetic exposure associations have been reported in **Supplemental Table S2 and Supplemental Table S3**.<br>b) The causal effect estimates between exposures, mediators and outcomes were listed in **Figure 2, 4**, **Supplemental Table S4**, and **Supplemental Table S5**. Our results were presented in terms of odds ratio and confidence intervals throughout the results section for binary outcomes and as beta coefficient for quantitative outcomes.

c) If relevant, consider translating estimates of relative risk into absolute risk for a meaningful time-period.<br>d) Consider any plots to visualize results (e.g. forest plot, scatter plot of associations between genetic variants and outcome versus between genetic variants and exposure).	
**12. Assessment of assumptions**<br>a) Assess the validity of the assumptions.<br>b) Report any additional statistics (e.g., assessments of heterogeneity, such as I2, Q statistic).	a) We assess the validity using sensitivity analyses, MR Egger regression, weighted median approach and MR-PRESSO approach. Results were presented in Results section<br>b) We presented the use of Egger intercept and Cochran's Q in the Results (**Page 12**), **Supplemental Table S4**, and **Supplemental Table S5**.
**13. Sensitivity and additional analyses**<br>a) Use sensitivity analyses to assess the robustness of the main results to violations of the assumptions.<br>b) Report results from other sensitivity analyses (e.g., replication study with different dataset, analyses of subgroups, validation of instrument(s),simulations, etc.).<br>c) Report any assessment of direction of causality (e.g., bidirectional MR).<br>d) When relevant, report and compare with estimates from non-MR analyses.<br>e) Consider any additional plots to visualize results(e.g., leave-one-out analyses).	a) we reported the use of additional independent data as additional approach to validate main results in Results (**Page 4**).
**Discussion**	
**14. Key results**	Discussion paragraph 1 and 2(**Page 8 and Page 9**)

**15. Limitations**<br>Discuss limitations of the study, taking into account the validity of the MR assumptions, other sources of potential bias, and imprecision. Discuss both direction and magnitude of any potential bias, and any efforts to address them.	Study limitations (**Page 10**)
**16. Interpretations**<br>a) Give a cautious overall interpretation of results considering objectives and limitations. Compare with results from other relevant studies.<br>b) Discuss underlying biological mechanisms that could be modelled by using the genetic variants to assess the relationship between the exposure and the outcome.<br>c) Discuss whether the results have clinical or policy relevance, and whether interventions could have the same size effect.	a) Interpretation: Discussion paragraphs 1, 2, 3, 4, 5 ,6(**Page 8 -10**)<br>b) Underlying biological mechanisms: Discussion paragraphs 2, 3, 4 (**Page 8** and **Page 9**).<br>c) Conclusion (**Page 10**)
**17. Generalizability:**	We have discussed the potential caveats in terms of generalizability of our findings in the Limitation section (**Page 10**).
**18. Funding:**	We have reported all sources of funding in the "Funding" section.
**19. Data and data sharing:**	We have provided the link/approach to access genetic data used in this study in the "Data Availability Statement" section.
**20. Conflicts of Interest:**	We have declared conflicts of interest in the "Disclosure" section.
